# Distribution of *Trichinella spiralis, Trichinella britovi*, and *Trichinella pseudospiralis* in the Diaphragms and *T. spiralis* and *T. britovi* in the Tongues of Experimentally Infected Pigs

**DOI:** 10.3389/fvets.2021.696284

**Published:** 2021-06-22

**Authors:** Michał Gondek, Przemysław Knysz, Renata Pyz-Łukasik, Anna Łukomska, Anna Kuriga, Małgorzata Pomorska-Mól

**Affiliations:** ^1^Department of Food Hygiene of Animal Origin, Faculty of Veterinary Medicine, University of Life Sciences in Lublin, Lublin, Poland; ^2^Department of Preclinical Sciences and Infectious Diseases, Faculty of Veterinary Medicine and Animal Science, Poznań University of Life Sciences, Poznań, Poland

**Keywords:** pig, *Trichinella* spp, diaphragm, tongue, larvae distribution, experimental infection, histopathology

## Abstract

There is little or even no data in the global literature on the distribution of different species of *Trichinella* in the individual parts of the diaphragms and tongues in infected pigs. This is of particular importance from the food safety point of view and for the conduct of routine testing of pig carcasses for *Trichinella* as well as epidemiological surveys. Therefore, the aim of the present study was to evaluate the distribution of *Trichinella spiralis (T. spiralis), Trichinella britovi (T. britovi)*, and *Trichinella pseudospiralis (T. pseudospiralis)* ML in various parts of the diaphragm (the pillars, costal, and sternal part) and the distribution of encapsulated species of *Trichinella* (*T. spiralis* and *T. britovi*) in various parts of the tongues (the tip, body, and root) of experimentally infected pigs. The diaphragm pillars were the most heavily parasitized part of the diaphragm both in groups of pigs infected with particular species of *Trichinella* and in groups of pigs presenting different levels of infection; however, statistical differences were observed only in the group of pigs with moderate (21–35 larvae per gram-lpg) or moderately high (35–55 lpg) intensity of *Trichinella* spp. infection in the entire diaphragm. In all groups of pigs, regardless of the infecting *Trichinella* species or infection level, larvae showed a homogeneous distribution on both sides of the diaphragm and excluding those of *T. pseudospiralis*, also in all three parts of the tongue. Histological examination showed features of a differential inflammatory response around larvae of the different *Trichinella* species. This study confirmed that for mandatory examination of pig carcasses using a pooled-sample digestion assay in which each pig is intended to be represented by a 1 gram sample taken from the diaphragm pillars, if that tissue is not available, the mass of the sample taken from the remaining diaphragm parts (costal or sternal) should be at least double that from the pillars. Histological findings confirmed that the inflammatory pattern of pig muscles varies depending on the *Trichinella* species triggering the infection and is less intense in the case of infections with *T. pseudospiralis* than in infections with encapsulated species of *Trichinella* (*T. spiralis* and *T. britovi*).

## Introduction

Nematodes of the genus *Trichinella* are the causative agent of a serious human zoonotic disease; namely, trichinellosis. Although the natural host range of *Trichinella* is extremely broad, including such taxonomic classes as mammals, birds and reptiles, from the public health protection point of view, pigs are the main concern because they play the pivotal role in the transmission of this pathogen to humans. Regardless of the host species or *Trichinella* species causing the infection, the parasite life cycle is the same: infection occurs after consumption of raw or undercooked meat or meat products infected with first-stage (L1) muscle larvae (ML) of *Trichinella*. Swallowed larvae are released from muscle tissue upon gastric digestion and subsequently reach the small intestine, where they penetrate intestinal mucosa, undergo four molts, and develop into dioecious adult worms. After mating, females start to release newborn larvae as early as 5–7 days post-infection (dpi) (1). The larvae penetrate intestinal walls, enter blood and lymphatic vessels, and migrate via the circulatory system to striated muscles, where they develop into fully infective ML. The target site for *Trichinella* is therefore striated muscles; however, the affinity of the parasite for individual muscles and/or muscle groups varies significantly. In fact, *Trichinella* larvae settle in the highest density in the muscles that exhibit the greatest functional activity and have the richest blood supply. It is obvious that the extensive blood supply of the muscles mechanically promotes a greater inflow of the larvae to them; however, other molecular mechanisms involved in the uneven distribution of *Trichinella* larvae in the musculature of the hosts are not fully elucidated. Muscles with the highest intensity of *Trichinella* larvae infection are termed predilection muscles or predilection sites, and the choice of a predilection site to study in terms of its anatomical location during a carcass examination depends solely on the host species. The *Trichinella* species inducing the infection also affects the larvae distribution; however, typically the examiner working on a carcass can only proceed on the basis of certainties, of which the identity of the infecting species is not one. Further, the time that has elapsed from infection to testing and thus the interim growth of animal and its musculature also plays a role. In this regard, changes in muscle weights reduce the larvae density and, because the growth kinetics of particular muscles vary, the proportionality in terms of larval load (calculated as number of larvae per gram of muscle—lpg) between different muscles may also change over the duration of the infection. In addition, the level of infection may also affect the distribution of the larvae in particular muscles of the host.

Numerous studies have been performed to identify the predilection sites for *Trichinella* larvae in pigs under experimental conditions; however, *T. spiralis* was the most frequently used in these experiments and other species are underrepresented. In pigs infected with *T. spiralis*, the diaphragm or tongue were identified as the most heavily parasitized muscles regardless of the dose, infection level, pig weight, or elapsed time from infection to slaughter; however, some significant differences between particular experimental trials have been observed. Prost and Nowakowski (2), for instance, showed that the mean intensity of *T. spiralis* infection in the tongue of pigs infected with this species was only 52% of that observed in the diaphragm. Similar observations were made by Korínková et al. (3), in which the mean infection intensity in the tongues of pigs experimentally infected with 500 *T. spiralis* ML achieved 39% of that detected in the diaphragm. Furthermore, Gondek et al. (4) found that the larval density in the tongues of pigs experimentally infected with 300 ML of *T. spiralis* was significantly lower as compared with this density in diaphragms and constituted only 53% of that found in the latter tissue type. However, when a higher infective dose was used (1,000 *T. spiralis*), the intensities of infection in the tongues and diaphragms of the infected pigs were comparable (5). Contrastingly, a study applying a comprehensive statistical approach proved that in the case of *T. spiralis* infection, it was the pig tongue which exhibited the statistically highest intensity of *Trichinella* larvae infection (6). Moreover, some reports also showed that the intensity of *T. spiralis* infection in the diaphragm of experimentally infected pigs amounted to only 26 or 33–37% of that observed in the tongue (7, 8). Recently, Wang et al. (9) found that the distribution of *T. spiralis* ML in the diaphragms and tongues of infected pigs may be dose-dependent; the diaphragm showed the highest larval burden in the case of infective doses below 400 ML, while tongue had the highest intensity of infection when higher doses (400–10,000) were used. Many additional studies demonstrated that the diaphragm and tongue might be considered equally valuable research material for *T. spiralis* muscle larvae detection; the intensities of infection of these two muscles were comparable in different experimental trials, but holistically, the diaphragm was more often ranked first (8, 10–18). It should also be emphasized that there is much less information on the larval preference for one of these two muscles in pigs infected with two additional *Trichinella* species which have also been confirmed to occur in the European pig population; namely, *T. britovi* and *T. pseudospiralis*. In this regard, the most relevant and comprehensive results were achieved by Kapel et al. (19), who reported that in pigs, the tongue and diaphragm were found to be predilection sites for all genotypes of *Trichinella*; however, in the case of encysted genotypes (*T. spiralis, T. nativa, T. britovi*, and *T. nelsoni*) the stronger predilection was for the tongue, while for the non-encapsulated ones (*T. pseudospiralis*) it was for the diaphragm.

Regardless of all these studies, there is little or even no data in the global literature on the distribution of different species of *Trichinella* in various parts of the diaphragms and tongues of infected pigs. This is of particular importance from the food safety point of view and for the conduct of routine testing of pig carcasses for *Trichinella* as well as epidemiological surveys. Only two studies performed by Kotula et al. (20) and Serrano et al. (21) provide deeper insight into the distribution of *Trichinella* larvae in particular parts and sides of the diaphragm, and both of these experiments did not yield results for a broad variety of species, applying only to pigs infected with *T. spiralis* or *T. spiralis*/*T. britovi* (as a single group). Moreover, in each of the research works referred to, the techniques of anatomical dissection of the individual parts of the diaphragm was different and, as a consequence, different sections of the diaphragm were subjected to examination. Therefore, the aim of the present study was to evaluate the distribution of *T. spiralis, T. britovi*, and *T. pseudospiralis* ML in various parts of the diaphragm of experimentally infected pigs. In this regard, we examined three different regions of the diaphragm (the diaphragm pillars, costal part and sternal part) which are listed in Commission Implementing Regulation (EU) 2015/1375, the main legal act regulating the examination of pigs for *Trichinella* in Europe (22). Moreover, the distribution of *Trichinella* larvae was assessed in relation to the diaphragm side. Two different variables were used in the statistical model construction; namely, the *Trichinella* species used for experimental pig infection and the level of *Trichinella* infection in the entire diaphragm of the infected pigs. In addition, the distribution of encapsulated species of *Trichinella* (*T. spiralis* and *T. britovi*) in various parts of the tongues (the tip, body and root) of infected pigs was also examined in this study.

## Materials and Methods

### Parasites, Pig Infection, Necropsy, and Dissection of Individual Parts of the Diaphragms and Tongues

Three different *Trichinella* species (genotypes), *Trichinella spiralis* (T1), *Trichinella britovi* (T3), and *Trichinella pseudospiralis* (T4), were used. *Trichinella spiralis* and *Trichinella britovi* ML were isolated in our laboratory during routine inspections of meat from a naturally infected pig and a wild boar, respectively. Subsequently, both of these isolates were identified at the species level by multiplex polymerase chain reaction (multiplex PCR) according to Zarlenga et al. (23). *Trichinella pseudospiralis* was a kind gift from the Witold Stefański Institute of Parasitology of the Polish Academy of Sciences.

The studies involved 27 young healthy Pulawska/Polish Large White crossbreed pigs (both sexes, aged 10–12 weeks, average body weight 24.4 ± 6.5 kg) purchased from a farm without any known *Trichinella* infection history. A serological test (Priocheck Trichinella Ab, Thermo Fisher, USA) was also performed to confirm the *Trichinella*-negative status of these pigs.

*Trichinella spiralis, T. britovi*, and *T. pseudospiralis* muscle larvae displaying motility were counted (each species separately) and then suspended in 30% gelatin blocks. Pigs were infected by administering a single dose *per os* of 300 *T. spiralis* ML per pig (group T1; *n* = 10); 800 *T. pseudospiralis* ML per pig (group T4; *n* = 9); and 5,000 *T. britovi* ML per pig (group T3; *n* = 8). Pigs were slaughtered at 21–62 days after experimental inoculation. The average (±SD) body weight of pigs on the day of slaughter was 32.8 ± 9.4 kg.

Immediately after the pigs were sacrificed, the abdominal integuments were cut along the midline (*linea alba*) and the stomach and intestines were removed. Then, the entire diaphragm was harvested from each carcass (Supplementary Figure 1A). Tendons, fasciae, fat, and serous membranes were discarded, leaving the diaphragm for examination (Supplementary Figure 1B). Subsequently, the diaphragm was dissected into the following parts: the pillars (*crura*), costal part (*pars costalis*), and sternal part (*pars sternalis*). The following anatomical relationships were considered during the dissection of the pig diaphragm into individual parts: (i) the pillars of the pig diaphragm are attached to the ventral surface of the first lumbar vertebrae; (ii) the costal part of the pig diaphragm begins dorsally at the level of the last rib on its inner side. Subsequently, it descends along the joints of the costal bones with their cartilages up to the eighth rib, from where, along the cartilage of this rib it turns into the sternal part of the diaphragm on the xiphoid process; (iii) the sternal part of the pig diaphragm attaches to the inner surface of the xiphoid process of the sternum. Moreover, the diaphragm and all its anatomical parts were divided into left and right sides according to the symmetry of the carcass. A detailed scheme of the pig diaphragm dissection is shown in Supplementary Figures 1A,B.

The tongue was completely removed from the oral cavity and dissected from the throat structures (epiglottis and pharyngeal inlet). Then, the tongues from each pig were divided into the following parts: the tip of the tongue (*apex linguae*—the narrow part which is directed forward against the lingual surface of the lower incisors), the body of the tongue (*corpus linguae*—the forward two-thirds that lie in front of the *sulcus terminalis*), and the root of the tongue (*radix linguae*—the back third attached to the hyoid bone by the hyoglossus and genioglossus muscles as well as the hyoglossal membrane). A detailed scheme of the pig tongue dissection is shown in Supplementary Figures 2A,B.

### Muscle Sample Digestion and Enumeration of Larvae

The muscle larvae intensity was determined by the digestion procedure laid down in European Commission Regulation EU 2015/1375 (22). Briefly, the individual muscle samples were minced in a blender and mixed with artificial digestive fluid [1,000 or 2,000 mL H_2_O, 8 or 16 mL of 25% HCl (Chempur, Poland), and 5 or 10 g of pepsin 2,000 FIP/g (BTL, Poland)]. The digestion step was carried out using a magnetic stirrer at 45° C (±1°C) for 30 (diaphragm samples) or 45 (tongue samples) min. After digestion, the digestion fluid was poured through a 180 μm sieve into a separation glass funnel. If more than 0.5 g of sample was retained in the sieve, the tissue was returned and the digestion procedure was repeated. The first sedimentation was carried out for 30 min. Subsequently, 40 mL of digestion fluid was run off into the centrifuge tube, and the second sedimentation was performed for 10 min. Supernatant in a 30 mL volume was removed by suctioning with a syringe needle. The remaining 10 mL of sediment was poured into a larval counting basin. The cylinder was rinsed with 10 mL of water and then the washings were added to the examined sample as well. A standard projection-trichinoscope (TPE) with a magnification of 50–80 × was used to count *T. spiralis, T. britovi*, or *T. pseudospiralis* ML. The intensity of *Trichinella* ML infection in the muscles was calculated as the number of larvae per gram (lpg) of muscle tissue. The individual parts of diaphragms and tongues were digested entirely. The average weights (±SD) of parts of the diaphragm in the group of pigs infected with *T. spiralis* were as follows: 16.92 ± 3.56 g (left pillar), 21.82 ± 3.51 g (right pillar), 21.98 ± 8.84 g (left costal part), 23.56 ± 9.21 g (right costal part), 6.12 ± 2.44 g (left sternal part), and 6.55 ± 2.58 g (right sternal part). For the *T. britovi*-infected group of pigs they were 20.96 ± 4.20 g (left pillar), 26.29 ± 5.05 g (right pillar), 30.06 ± 7.35 g (left costal part), 31.52 ± 7.33 g (right costal part), 7.61 ± 1.30 g (left sternal part), and 7.96 ± 1.13 g (right sternal part). The corresponding values for the *T. pseudospiralis*-infected group of pigs were as follows: 15.47 ± 3.73 g (left pillar), 18.23 ± 3.82 g (right pillar), 14.46 ± 3.52 g (left costal part), 16.51 ± 3.77 g (right costal part), 4.76 ± 0.90 g (left sternal part), and 4.83 ± 1.47 g (right sternal part). The average weights (±SD) of individual parts of the tongue in pigs infected with *T. spiralis* were as follows: 10.77 ± 2.57 g (tip), 35.04 ± 7.37 g (base), and 21.86 ± 3.31 g (root). For *T. britovi*-infected pigs they were 12.90 ± 1.85 g (tip), 41.71 ± 6.87 g (base), and 24.84 ± 2.79 g (root).

### Histological Examination of Pig Diaphragm Muscles

Diaphragm tissue samples were taken from six individuals from each experimental group of pigs at 60–62 dpi. Samples were fixed in neutral 10% formalin solution and then prepared according to the routine paraffin method. Paraffin sections 3 μm thick stained with hematoxylin (Gills) and eosin (H&E) were analyzed and micrographs were taken by using an Axio Lab.A1 microscope (Carl Zeiss, Germany) equipped with an ERc5s digital camera (Carl Zeiss). ZEN 2.3 (blue edition) software (Carl Zeiss) were used in these analyses.

### PCR Analysis to Confirm Species Identity of *Trichinella* in the Muscles of Infected Pigs

In order to confirm that the larvae of *Trichinella* isolated from diaphragms of the infected pigs were actually *T. spiralis, T. britovi*, or *T. pseudospiralis*, 50 larvae from each pig were pooled and then identified by multiplex PCR according to the method described by Zarlenga et al. (23). Larvae from pigs infected with *T. spiralis* were pooled from two individuals, larvae isolated from pigs infected with *T. britovi* were analyzed individually for each pig, as were larvae isolated from five pigs infected with *T. pseudospiralis*, while larvae from the remaining four pigs were pooled from two individuals and then analyzed. As a positive control, the following reference strains were used: *T. spiralis* ISS003, *T. nativa* ISS042, *T. britovi* ISS002, and *T. pseudospiralis* ISS013.

### Statistical Analysis

Data are presented as means and standard deviations. Medians and interquartile ranges are also included. The obtained data were tested for normality with a Shapiro–Wilk test and for homogeneity of variances with Levene's test. In order to compare the intensity of infection in the left and right side of the individual diaphragm parts (i.e., left pillar vs. right pillar; left costal part vs. right costal part; and left sternal part vs. right sternal part) as well as the left and right hemidiaphragms, Student's *t*-test (all assumptions met) or the Mann–Whitney *U*-test (for non-normally distributed data) was used. In order to compare the intensity of infection in the three different parts of the diaphragm (i.e., pillars vs. costal part vs. sternal part) the following statistical tests were performed: one-way analysis of variance (ANOVA) with a *post-hoc* Tukey test (all assumptions met) or non-parametric Kruskal–Wallis test (for non-normally distributed data). Two different statistical models were used in these analyses. In the first, pigs were divided into three groups (*T. spiralis, T. britovi*, and *T. pseudospiralis*) according to the species of *Trichinella* that was used for infection. In the second one, pigs were sorted into five groups depending on the level of infection in the entire diaphragm (group I: 4–12 lpg; group II: 12–21 lpg; group III: 21–35 lpg; group IV: 35–55 lpg; and group V: > 60 lpg).

The three different anatomical parts of the tongue (i.e., the tip, body, and root) in a specific group of swine infected with encapsulated species of *Trichinella* (*T. spiralis* and *T. britovi*) were compared with each other. In this analysis, the following statistical operations were applied: one-way analysis of variance with Welch's correction (datasets that exhibited normality but did not exhibit homogeneity of variances) or the non-parametric Kruskal–Wallis test (for non-normally distributed data).

Finally, correlations between the lpg of different parts of the diaphragm and the lpg of different sections of the tongue within the group of pigs infected with a particular species of *Trichinella* were calculated using Pearson's (normally distributed data) or Spearman's (non-normally distributed data) correlation as appropriate.

For all these analyses, the level of significance was set at *P* < 0.05. All statistical calculations were performed with Statistica software (StatSoft, Poland).

## Results

### Intensity of *T. spiralis, T. britovi*, and *T. pseudospiralis* ML Infection and Distribution of the Larvae in Various Parts of the Diaphragm in the Groups of Experimentally Infected Pigs

Statistically compiled results regarding the intensity of *T. spiralis, T. britovi*, and *T. pseudospiralis* ML infection (lpg) and distribution of the larvae in particular parts of the diaphragm within the groups of pigs each experimentally infected with only one of the three *Trichinella* species are shown in Tables 1–3.

**Table 1:** Intensity of *Trichinella spiralis* ML infection (lpg) and distribution of the larvae in the different parts of the diaphragm of pigs (*n* = 10) experimentally infected with 300 ML of *T. spiralis*.

**Part of the diaphragm**		**Intensity of** ***T. spiralis*** **ML infection (lpg)**			***P* (left side vs. right side)**
		**Side**			
		**Left**	**Right**	**Left and right (combined)**	
Pillars (*crura*)	Mean	54.59	53.25	53.87	0.934*
	SD	35.78	35.10	35.12	
	Median	59.83	47.92	53.40	
	IQR	31.06–74.10	29.79–82.01	30.33–78.47	
Costal part (*pars costalis*)	Mean	40.64	39.95	40.27	0.961*
	SD	31.65	30.15	30.64	
	Median	32.17	40.80	37.75	
	IQR	23.42–60.61	13.73–59.36	18.42–60.00	
Sternal part (*pars sternalis*)	Mean	40.13	44.62	42.56	0.761*
	SD	32.28	32.81	32.50	
	Median	31.28	38.08	35.75	
	IQR	18.91–63.05	21.10–78.08	20.07–72.03	
*P* (pillars vs. costal part vs. sternal part)		0.552**	0.658**	0.616**	-
Diaphragm (*diaphragma*) (entire)	Mean	46.02	46.31	46.18	0.985*
	SD	33.32	32.79	32.96	
	Median	43.39	44.68	44.10	
	IQR	26.86–68.74	21.90–72.58	24.32–70.86	

The left side of each anatomical part of the diaphragm was compared with the right side (marked in the table as: left side vs. right side). Individual parts of the diaphragm (the pillars, costal part, and sternal part) of each side (left, right and both together) were compared with each other (marked in the table as “pillars vs. costal part vs. sternal part”). P-values were calculated using Student's t-test (*) and one-way ANOVA (**). SD, standard deviation; IQR, interquartile range (Q25–Q75%).

**Table 2:** Intensity of *Trichinella britovi* ML infection (lpg) and distribution of the larvae in the different parts of the diaphragm of pigs (*n* = 8) experimentally infected with 5,000 ML of *T. britovi*.

**Part of the diaphragm**		**Intensity of** ***T. britovi*** **ML infection (lpg)**			***P* (left side vs. right side)**
		**Side**			
		**Left**	**Right**	**Left and right (combined)**	
Pillars (*crura*)	Mean	44.20	43.26	43.67	1.00**
	SD	17.91	16.05	16.57	
	Median	47.27	50.56	49.32	
	IQR	31.78–55.33	30.12–55.46	31.33–55.41	
Costal part (*pars costalis*)	Mean	30.35	33.51	31.95	0.608*
	SD	12.05	12.03	11.59	
	Median	32.97	37.13	32.87	
	IQR	17.35–40.43	23.41–41.69	22.62–41.09	
Sternal part (*pars sternalis*)	Mean	39.48	36.12	37.61	0.664*
	SD	16.45	13.63	14.50	
	Median	33.65	37.97	35.80	
	IQR	27.20–57.88	24.68–47.64	23.15–52.61	
*P* (pillars vs. costal part vs. sternal part)		0.223***	0.203****	0.285***	-
Diaphragm (*diaphragma*) (entire)	Mean	36.04	37.54	36.83	0.817*
	SD	12.08	13.44	12.78	
	Median	39.67	41.74	40.75	
	IQR	25.30–45.33	25.97–48.03	25.63–46.76	

The left side of each part of the diaphragm was compared with the right side (marked in the table as “left side vs right side”). Individual parts of the diaphragm (the pillars, costal part, and sternal part) of each side (left and right and both together) were compared with each other (marked in the table as “pillars vs costal part vs. sternal part”). P-values were calculated using Student's t-test (*) or Mann–Whitney U-test (**), and one-way ANOVA (***) or Kruskal–Wallis test (****). SD, standard deviation; IQR, interquartile range (Q25–Q75%).

**Table 3:** Intensity of *Trichinella pseudospiralis* ML infection (lpg) and distribution of the larvae in the different parts of the diaphragm of pigs (*n* = 9) experimentally infected with 800 ML of *T. pseudospiralis*.

**Part of the diaphragm**		**Intensity of** ***T. pseudospiralis*** **ML infection (lpg)**			***P* (left side vs. right side)**
		**Side**			
		**Left**	**Right**	**Left and right (combined)**	
Pillars (*crura*)	Mean	27.09	26.71	26.92	0.929**
	SD	25.79	27.19	26.42	
	Median	16.48	15.89	14.89	
	IQR	9.29–33.08	9.15–33.41	9.22–33.25	
Costal part (*pars costalis*)	Mean	24.16	22.66	23.32	0.891*
	SD	24.00	21.67	22.65	
	Median	16.30	14.91	14.85	
	IQR	7.58–28.36	7.05–30.95	7.30–29.71	
Sternal part (*pars sternalis*)	Mean	22.34	19.85	21.10	0.757**
	SD	24.36	21.33	22.67	
	Median	14.62	10.00	12.32	
	IQR	10.43–24.13	7.48–19.67	8.78–21.85	
*P* (pillars vs. costal part vs. sternal part)		0.887***	0.742***	0.777***	-
Diaphragm (*diaphragma*) (entire)	Mean	25.22	24.20	24.67	0.930**
	SD	24.78	24.09	24.31	
	Median	15.48	14.83	14.61	
	IQR	8.75–29.93	8.07–30.67	8.39–30.31	

The left side of each part of the diaphragm was compared with the right side (marked in the table as “left side vs right side”). Individual parts of the diaphragm (the pillars, costal part and sternal part) of each side (left and right and both together) were compared with each other (marked in the table as “pillars vs costal part vs sternal part”). P-values were calculated using Student's t-test (*) or Mann–Whitney U-test (**) and Kruskal–Wallis test (***). SD, standard deviation; IQR, interquartile range (Q25–Q75%).

Muscle larvae of all *Trichinella* genotypes used in this study showed a homogeneous distribution on both sides of the diaphragm since no statistically significant differences in larvae density were found between the left and right hemidiaphragms in pigs infected with *T. spiralis* (Student's *t*-test, *P* = 0.985), *T. britovi* (Student's *t*-test, *P* = 0.817) or *T. pseudospiralis* (Mann–Whitney *U*-test, *P* = 0.930). Similarly, in all three groups of pigs, there were no significant differences in the intensity of infection between the left and right diaphragm pillars (Student's *t*-test, *P* = 0.934; Mann–Whitney *U*-test, *P* = 1.00; and Mann–Whitney *U*-test, *P* = 0.929 for *T. spiralis*-, *T. britovi*- and *T. pseudospiralis*-infected pigs, respectively), left and right costal parts (Student's *t*-test, *P* = 0.961; *P* = 0.608; and *P* = 0.891 for the respective groups) or left and right sternal parts of the diaphragm (Student's *t*-test, *P* = 0.761; Student's *t*-test, *P* = 0.664; and Mann–Whitney *U*-test, *P* = 0.757, respectively).

Neither were statistically significant differences in terms of intensity of *Trichinella* ML infection found between the three different parts of the diaphragm, i.e., the pillars, costal, and sternal part, in the groups of pigs infected with *T. spiralis* (ANOVA, *P* = 0.616), *T. britovi* (ANOVA, *P* = 0.285), or *T. pseudospiralis* (Kruskal–Wallis, *P* = 0.777). Moreover, these three parts did not differ from each other on the left and right side in all three groups of infected pigs (ANOVA or Kruskal–Wallis, *P* > 0.05). Although statistical differences were not demonstrated, the diaphragm pillars were the most heavily parasitized part of the diaphragm in all three groups of pigs. It was particularly salient in the group of pigs infected with *T. spiralis*, where the intensity of infection in the diaphragm pillars (53.87 lpg) was, respectively, 134 and 127% of that observed in the costal and sternal parts while in *T. britovi*-infected pigs the respective values were 137 and 116% for intensity in the pillars of 43.67 lpg. Furthermore, as shown in Table 4, out of a total of 27 pigs experimentally infected with three different genotypes of *Trichinella*, in 23 individuals (85.19%) the diaphragm pillars presented the highest intensity of infection. These tissue sections were ranked first for intensity in 9 out of 10 (90%) pigs infected with *T. spiralis*, 5 out of 8 (62.5%) pigs infected with *T. britovi* and in all nine pigs (100%) infected with *T. pseudospiralis*. The sternal part of the diaphragm was most infected in four (14.81%) pigs (one pig infected with *T. spiralis* and three pigs infected with *T. britovi*), while the costal part of the diaphragm was second most infected (in 14 pigs, which was 51.85% of all those infected) or third (in 12 pigs, which was 44.44% of all those infected) (Table 4). In the group of pigs (excluding pig no. 26 with only one larva in one diaphragm pillar) in which the diaphragm pillars revealed the highest intensity of *Trichinella* ML infection, the average larval density in the costal part was 76.31% (range: 44.59–99.73%) and in the sternal part 75.10% (range: 40.68–97.33%) of that observed in the pillars. However, the costal part of the diaphragm had 72.09% (range: 53.94–88.26%), 69.83% (range: 44.59–89.42%), and 85.10% (range: 55.08–99.73%) of the infection density seen in the pillars in pigs infected with *T. spiralis, T. britovi*, and *T. pseudospiralis*, respectively. These values calculated for the sternal part of the diaphragm were rather similar and amounted to 75.06% (range: 58.9–97.33%), 76.86% (range: 51.29–97.28%), and 74.04% (40.68–96.03%) for *T. spiralis, T. britovi*, and *T. pseudospiralis*, respectively. Table 5 summarizes the correlations between the lpg of different parts of the diaphragm within the group of pigs infected with *T. spiralis* (T1), *T. britovi* (T3), and *T. pseudospiralis* (T4). In the group of pigs infected with encapsulated species of *Trichinella*, a significant positive correlation was found between the lpg values of the pillars and costal part (*R* = 0.980 and *R* = 739 for pigs infected with *T. spiralis* and *T. britovi*, respectively); pillars and sternal part (*R* = 0.967 and *R* = 0.750 for pigs infected with *T. spiralis* and *T. britovi*, respectively); and costal and sternal part (*R* = 0.973 and *R* = 0.730 for the respective groups of pigs). In the group of pigs infected with *T. pseudospiralis*, a strong positive correlation was found between the lpg values of the pillars and costal parts (*R* = 0.983) and between those of the pillars and sternal parts (*R* = 0.983).

**Table 4:** Individual ranking of the three different parts of the diaphragm of pigs experimentally infected with *T. spiralis* (300 ML/pig), *T. britovi* (5,000 ML/pig) or *T. pseudospiralis* (800 ML/pig) in terms of the density of *Trichinella* ML.

**Pig no**.	**Intensity of infection of the entire diaphragm (lpg)**	**Ranking of the parts of the diaphragm in terms of lpg value^a^** **(Percentage calculated by using the diaphragm part with the highest number of lpg as the reference equal to 100%)**			**Pig diaphragm mass on the day of slaughter (g)**	***Trichinella* species used for experimental infection**
		**Pillars (*crura*)**	**Costal part (*pars costalis*)**	**Sternal part (*pars sternalis*)**		
26	0.01^b^	**1 (100)**	ND (0)	ND (0)	69.85	*T. pseudospiralis*
1	4.26	2 (83.66)	3 (63.20)	**1 (100)**	102.80	*T. spiralis*
24	5.25	**1 (100)**	2 (55.08)	3 (40.68)	94.70	*T. pseudospiralis*
20	8.39	**1 (100)**	3 (79.18)	2 (95.23)	94.30	*T. pseudospiralis*
10	11.94	**1 (100)**	3 (69.73)	2 (77.25)	67.25	*T. spiralis*
19	13.36	**1 (100)**	2 (98.02)	3 (66.83)	70.50	*T. pseudospiralis*
21	14.61	**1 (100)**	2 (99.73)	3 (82.74)	77.75	*T. pseudospiralis*
16	17.91	2 (91.92)	3 (70.84)	**1 (100)**	167.25	*T. britovi*
15	18.30	3 (74.20)	2 (80.24)	**1 (100)**	97.25	*T. britovi*
23	20.81	**1 (100)**	2 (88.36)	3 (72.66)	93.90	*T. pseudospiralis*
2	24.32	**1 (100)**	3 (53.94)	2 (62.28)	134.00	*T. spiralis*
3	26.35	**1 (100)**	2 (81.77)	3 (66.17)	117.20	*T. spiralis*
22	30.31	**1 (100)**	2 (89.35)	3 (65.71)	73.30	*T. pseudospiralis*
11	32.96	**1 (100)**	2 (61.87)	3 (51.29)	135.40	*T. britovi*
18	39.74	**1 (100)**	2 (89.42)	3 (88.60)	103.10	*T. britovi*
17	41.75	**1 (100)**	3 (44.59)	2 (87.97)	131.15	*T. britovi*
4	43.27	**1 (100)**	3 (67.16)	2 (76.67)	97.25	*T. spiralis*
12	44.32	**1 (100)**	2 (65.56)	3 (59.16)	116.80	*T. britovi*
6	44.92	**1 (100)**	2 (74.18)	3 (58.90)	69.85	*T. spiralis*
14	49.19	2 (94.59)	3 (78.66)	**1 (100)**	139.65	*T. britovi*
5	49.64	**1 (100)**	2 (59.88)	3 (59.23)	148.90	*T. spiralis*
13	50.45	**1 (100)**	3 (87.71)	2 (97.28)	104.60	*T. britovi*
27	63.72	**1 (100)**	2 (78.59)	3 (72.40)	68.60	*T. pseudospiralis*
25	65.61	**1 (100)**	3 (92.51)	2 (96.03)	55.80	*T. pseudospiralis*
7	70.86	**1 (100)**	3 (79.42)	2 (91.79)	86.25	*T. spiralis*
8	71.49	**1 (100)**	3 (74.50)	2 (97.33)	82.35	*T. spiralis*
9	114.75	**1 (100)**	2 (88.26)	3 (85.93)	63.60	*T. spiralis*

*The ranking data are cross-referenced with the infection level in the entire diaphragm, entire diaphragm mass, and Trichinella species triggering the infection*.

a *Diaphragm parts are ranked from the highest to the lowest lpg value. The part of the diaphragm with the highest lpg value is marked in bold*.

b *Only one T. pseudospiralis muscle larva was detected in the left diaphragm pillar. No larvae were detected in the costal or sternal parts of the diaphragm*.

*ND, not detected*.

**Table 5:** Correlations between the number of larvae per gram (lpg) of the different parts of the diaphragm in the groups of pigs infected with *T. spiralis* (300 ML/pig), *T. britovi* (5,000 ML/pig) and *T. pseudospiralis* (800 ML/pig).

**Diaphragm part**	**Diaphragm part**									
		**Pillars (*****crura*****)**			**Costal part (*****pars costalis*****)**			**Sternal part (*****pars sternalis*****)**		
		**T1**	**T3**	**T4**	**T1**	**T3**	**T4**	**T1**	**T3**	**T4**
Pillars (*crura*)	T1	-	-	-	0.980^*^	-	-	0.967^*^	-	-
	T3	-	-	-	-	0.739^*^	-	-	0.750^*^	-
	T4	-	-	-	-	-	0.983^*^	-	-	0.983^*^
Costal part (*pars costalis*)	T1	0.980^*^	-	-	-	-	-	0.973^*^	-	-
	T3	-	0.739^*^	-	-	-	-	-	0.730^*^	-
	T4	-	-	0.983^*^	-	-	-	-	-	1.00
Sternal part (*pars sternalis*)	T1	0.967^*^	-	-	0.973^*^	-	-	-	-	-
	T3	-	0.750^*^	-	-	0.730^*^	-	-	-	-
	T4	-	-	0.983^*^	-	-	1.00	-	-	-

*Data are R correlation coefficients, calculated using Pearson's or Spearman's correlation as appropriate*.

* *Correlation is significant at the 0.05 level*.

*T1, T. spiralis-infected pigs; T3, T. britovi-infected pigs; T4,T. pseudospiralis-infected pigs*.

### Distribution of *Trichinella* ML in Particular Parts of the Diaphragm in Pigs Grouped by Level of *Trichinella* ML Infection

Five groups of pigs with different larval loads in the entire diaphragm were constructed. Group I presented a low level of infection (4–12 lpg), group II a moderately low level (12–21 lpg), group III a moderate level (21–35 lpg), group IV a moderately high level (35–55 lpg), and group V showed a high infection level (>60 lpg).

**Figure 1:**
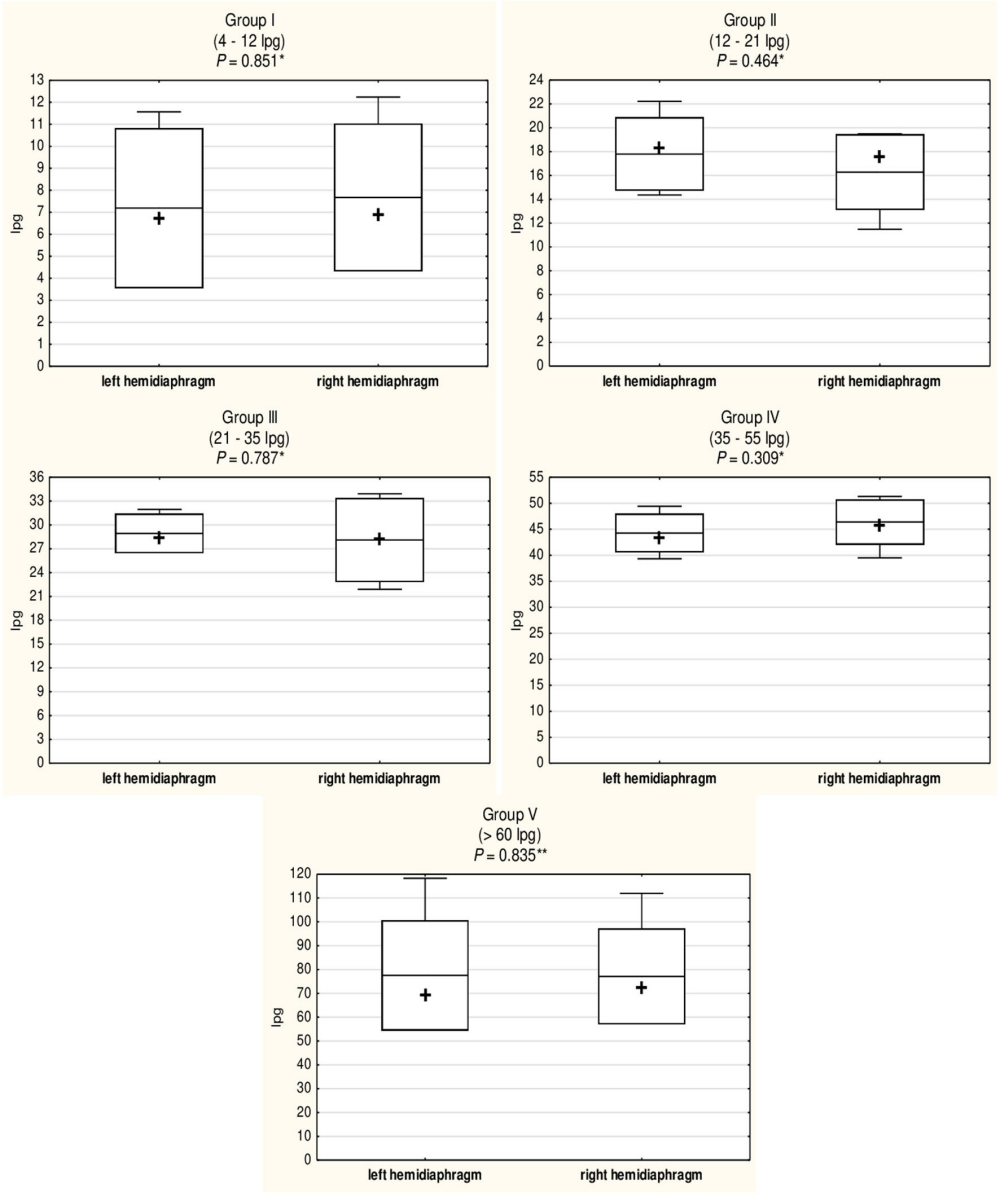
Comparison of the density of *Trichinella* spp. (*T. spiralis, T. britovi*, and *T. pseudospiralis*) ML in the left and right hemidiaphragm of five groups of experimentally infected pigs with different whole-diaphragm levels of infection. Group I, two hosts infected with *T. spiralis* and two hosts infected with *T. pseudospiralis*; Group II, two hosts infected with *T. britovi* and three hosts infected with *T. pseudospiralis*; Group III, two hosts infected with *T. spiralis*, one host infected with *T. britovi*, and one host infected with *T. pseudospiralis*; Group IV, three hosts infected with *T. spiralis* and five hosts infected with *T. britovi*; Group V, three hosts infected with *T. spiralis* and two hosts infected with *T. pseudospiralis*. Pigs were experimentally inoculated with 300 ML of *T. spiralis*, 5000 ML of *T. britovi*, or 800 ML of *T. pseudospiralis*. Box and whisker plots include means (line), medians (cross), standard deviations ± mean (upper and lower bar), and maximum and minimum values (whiskers). *P-*values were calculated using Student's *t*-test (*) or Mann–Whitney *U*-test (**).

In all five groups of pigs, regardless of the *Trichinella* ML infection level, larvae showed a homogeneous distribution on both sides of the diaphragm. As shown in Figure 1, no statistically significant differences in larval density were found between the left and right hemidiaphragm in pigs presenting low (Student's *t*-test, *P* = 0.851), moderately low (Student's *t*-test, *P* = 0.464), moderate (Student's *t*-test, *P* = 0.787), moderately high (Student's *t*-test, *P* = 0.309), or high (Mann–Whitney *U*-test, *P* = 0.835) whole-diaphragm *Trichinella* spp. infection levels. Similarly in all five groups, there were no significant differences in terms of intensity of *Trichinella* ML infection between the left and right diaphragm pillar (Student's *t*-test, *P* = 0.706; Student's *t*-test, *P* = 0.534; Student's *t*-test, *P* = 0.977; Student's *t*-test, *P* = 0.387; and Mann–Whitney *U*-test, *P* = 0.676 for respective groups in ascending order of infection level), left and right costal part (Student's *t*-test, *P* = 0.984; *P* = 0.661; *P* = 0.516; *P* = 0.136; and Mann–Whitney *U*-test, *P* = 0.296 for the respective groups of pigs) as well as left and right sternal part of the diaphragm (Student's *t*-test, *P* = 0.829; *P* = 0.390; Mann–Whitney *U*-test, *P* = 0.194; *P* = 0.713; and Student's *t*-test, *P* = 0.939) (Table 6). Furthermore, no statistically significant differences in larval density were found between the three different parts of the diaphragm (pillars, costal and sternal) in the groups of pigs presenting low (ANOVA, *P* = 0.590), moderately low (ANOVA, *P* = 0.875) or high (Kruskal–Wallis, *P* = 0.179) levels of *Trichinella* spp. infection (Table 6). In the groups of pigs with moderate or moderately high whole-diaphragm intensity of *Trichinella* ML infection, the larval density in the pillars was statistically significantly higher than that in the costal or sternal parts of the diaphragm. The values from ANOVA analysis were *F* = 8.98 and *P* = 0.007 for the moderately infected and *F* = 10.01 and *P* = 0.001 for the moderately high infected pigs, and the Tukey *post-hoc* test values presented as *P* = 0.03 (diaphragm pillars vs. costal part) and *P* = 0.02 (diaphragm pillars vs. sternal part) for pigs with *Trichinella* infection intensity in the 21–35 lpg range and *P* = 0.001 (diaphragm pillars vs. costal part) and *P* = 0.01 (diaphragm pillars vs. sternal part) for pigs with infection in the 35–55 lpg range (Table 6).

**Table 6:** Distribution of *Trichinella* spp. (*T. spiralis, T. britovi*, and *T. pseudospiralis*) ML in the different parts of the diaphragm of five groups of pigs with different levels of infection.

**Infection level in the entire diaphragm (group)**	**No. of pigs (*n*)**		**Intensity of** ***Trichinella*** **ML infection in individual parts of the diaphragm (lpg)**									***P*^**b**^**
			**Pillars**			**Costal part**			**Sternal part**			
			**Left**	**Right**	**Left and right (entire)**	**Left**	**Right**	**Left and right (entire)**	**Left**	**Right**	**Left and right (entire)**	
4–12 lpg (group I)	4	Mean	8.13	9.29	**8.80**	6.15	6.19	**6.16**	7.39	6.79	**7.06**	0.590***
		SD	4.33	3.94	**4.08**	3.24	2.87	**3.03**	4.24	3.11	**3.57**	
		Median	7.93	8.33	**8.15**	5.64	5.74	**5.60**	8.07	6.46	**7.18**	
		Q25%	4.89	6.60	**5.87**	3.48	3.94	**3.71**	3.95	4.46	**4.23**	
		Q75%	11.37	11.98	**11.73**	8.81	8.45	**8.62**	10.83	9.13	**9.89**	
		*P*^a^	0.706*		-	0.984*		**-**	0.829*		**-**	
12–21 lpg (group II)	5	Mean	18.65	16.96	**17.78**	16.98	16.16	**16.54**	18.91	14.72	**16.60**	0.875***
		SD	4.89	3.12	**3.64**	2.07	3.49	**2.64**	8.67	5.59	**5.82**	
		Median	16.48	17.57	**16.97**	16.30	14.91	**15.43**	15.04	15.36	**16.61**	
		Q25%	16.29	15.89	**14.89**	16.22	14.69	**14.85**	14.62	10.00	**12.32**	
		Q75%	20.14	19.29	**20.02**	17.32	19.34	**18.35**	21.76	18.11	**21,76**	
		*P*^a^	0.534*		**-**	0.661*		**-**	0.390*		**-**	
21–35 lpg (group III)	4	Mean	35.79	35.94	**35.85**^**A**^	26.68	23.97	**25.30**^**B**^	20.47	22.85	**21.65**^**B**^	0.007***
		SD	5.41	7.98	**6.74**	2.56	7.44	**5.02**	2.45	3.47	**1.39**	
		Median	34.35	33.15	**33.70**	27.13	25.60	**26.53**	19.42	22.14	**21.56**	
		Q25%	32.07	31.34	**31.79**	24.66	18.73	**21.61**	19.13	20.34	**20.67**	
		Q75%	39.52	40.54	**39.91**	28.70	29.21	**28.98**	21.81	25.37	**22.64**	
		*P*^a^	0.977*		**-**	0.516*		**-**	0.194**		**-**	
35–55 lpg (group IV)	8	Mean	57.20	53.38	**55.11**^**A**^	35.80	41.40	**38.68**^**B**^	42.86	43.37	**43.13**^**B**^	0.001***
		SD	8.10	9.01	**7.07**	9.07	4.28	**6.01**	12.96	6.91	**9.33**	
		Median	57.12	54.13	**53.65**	37.37	40.80	**38.96**	35.96	44.77	**40.32**	
		Q25%	50.53	47.92	**53.06**	32.17	38.22	**36.59**	33.04	37.97	**35.80**	
		Q75%	64.11	56.17	**58.73**	42.41	44.05	**42.20**	57.88	47.64	**52.61**	
		*P*^a^	0.387*		**-**	0.136*		**-**	0.713**		**-**	
> 60 lpg (group V)	5	Mean	83.34	85.26	**84.40**	72.81	67.77	**70.05**	73.86	74.93	**74.68**	0.179****
		SD	24.43	20.18	**21.69**	22.00	20.71	**21.25**	21.68	19.46	**19.44**	
		Median	74.10	82.01	**78.47**	65.02	59.36	**62.32**	73.47	78.08	**72.03**	
		Q25%	71.71	77.92	**72.82**	60.61	58.61	**60.00**	63.05	56.57	**65.26**	
		Q75%	78.21	82.13	**80.54**	68.36	60.00	**62.87**	73.95	83.47	**78.39**	
		*P*^a^	0.676**		-	0.296**		-	0.939*		-	

The left side of each part of the diaphragm was compared with the right side (statistical results are given in rows marked as P-value^a^). Individual parts of the diaphragm (the entire pillars, entire costal part and entire sternal part; shown in bold) were compared with each other (statistical results are given in column marked as P-value^b^). P-values were calculated using Student's t-test (*) or Mann–Whitney U-test (**) and one-way ANOVA (***) followed by Tukey HSD post-hoc test or Kruskal–Wallis test (****). Values marked in bold in the same row and with the same uppercase letter indicate no significant differences between individual parts of the diaphragm (the entire pillars, entire costal part and entire sternal part), whereas different uppercase letters (A,B) indicate significant differences between parts (P < 0.05). Group I, two hosts infected with T. spiralis and two hosts infected with T. pseudospiralis; Group II, two hosts infected with T. britovi and three hosts infected with T. pseudospiralis; Group III, two hosts infected with T. spiralis, one host infected with T. britovi, and one host infected with T. pseudospiralis; Group IV, three hosts infected with T. spiralis and five hosts infected with T. britovi; Group V, three hosts infected with T. spiralis and two hosts infected with T. pseudospiralis. Pigs were experimentally inoculated with 300 ML of T. spiralis, 5,000 ML of T. britovi, or 800 ML of T. pseudospiralis. SD, standard deviation; Q25%, 25th percentile; Q75%,75th percentile.

### Intensity of *T. spiralis* and *T. britovi* ML Infection and Distribution of the Larvae in Various Anatomical Parts of the Tongue in Experimentally Infected Pigs

Statistically compiled results regarding the intensity of infection (lpg) of the encapsulated *Trichinella* species (*T. spiralis* and *T. britovi* ML) and distribution of the larvae in individual parts of the tongue in pigs each experimentally infected with only one of these species are shown in Table 7. The muscle larvae of *T. spiralis* and *T. britovi* showed a homogeneous distribution in all three parts of the tongue since no statistically significant differences in terms of intensity of larvae infection were found between the tip, body and root of the tongue in pigs infected with *T. spiralis* (Kruskal–Wallis, *P* = 0.703) or *T. britovi* (ANOVA with Welch's correction, *P* = 0.211).

**Table 7:** Intensity of *T. spiralis* and *T. britovi* ML infection (lpg) and distribution of the larvae in the different parts of the tongue of pigs experimentally infected with 300 ML of *T. spiralis* (*n* = 10) and 5,000 ML of *T. britovi* (*n* = 8).

**Anatomical part of the tongue**		**Intensity of** ***Trichinella*** **ML infection (lpg) in pigs infected with:**	
		***T. spiralis***	***T. britovi***
Tip (*apex linguae*)	Mean	22.94	22.93
	SD	19.90	7.93
	Median	15.99	24.45
	IQR	14.11–30.55	16.21–27.09
Body (*corpus linguae*)	Mean	24.85	33.57
	SD	14.22	15.62
	Median	28.41	35.40
	IQR	15.13–34.99	18.08–47.52
Root (*radix linguae*)	Mean	23.66	29.02
	SD	24.79	10.75
	Median	18.70	30.84
	IQR	9.05–26.32	18.60–38.74
	*P*	0.703**	0.211*
Entire tongue (*lingua*)	Mean	24.30	30.34
	SD	16.41	11.42
	Median	24.18	33.72
	IQR	13.86–29.47	18.97–39.97

*Individual parts of the tongue (the tip, body and root) were compared with each other*.

P-values were calculated using one-way ANOVA with Welch's correction (*) or non-parametric Kruskal–Wallis test (**). SD, standard deviation; IQR, interquartile range (Q25–Q75%).

Out of a total of 18 pigs experimentally infected with *T. spiralis* and *T. britovi*, in five individuals (27.78%), the tip of the tongue presented the highest intensity of infection (Table 8). The intensity was greatest in the body of the tongue in nine (50%) pigs (in five pigs infected with *T. spiralis* and four pigs infected with *T. britovi*), while the root of the tongue was characterized by the highest larval load in four individuals (22.22%) (Table 8). In general, the intensity of infection in the less-infected parts was on average 67.82% (range: 20.85–94.45%) of the intensity in the most-infected part for both groups of pigs. By individual species, the values were 64.79% (range: 20.85–94.34%) for *T. spiralis* infection and 71.61% (27–94.45%) for *T. britovi* (Table 8). Table 9 summarizes the correlations between the number of larvae per gram (lpg) of different parts of the tongue in the groups of pigs infected with *T. spiralis* (T1) and *T. britovi* (T3). In the group of pigs infected with both *T. spiralis* and *T. britovi*, a significant positive correlation was shown between the intensities of infection in the body and root of the tongue (*R* = 0.830 and *R* = 0.942 for pigs infected with *T. spiralis* and *T. britovi*, respectively).

**Table 8:** Individual ranking of the three different parts of the tongues of pigs experimentally infected with *T. spiralis* (300 ML/pig) or *T. britovi* (5,000 ML/pig) in terms of the density of *Trichinella* ML.

**Pig no**.	**Intensity of infection of the entire tongue (lpg)**	**Ranking of the individual parts of the tongue in terms of lpg value^a^** **(Percentage calculated by using the part of the tongue with the highest number of lpg as the reference equal to 100%)**			**Pig tongue mass on the day of slaughter (g)**	***Trichinella* species used for experimental infection**
		**Tip (*apex linguae*)**	**Body (*corpus linguae*)**	**Root (*radix linguae*)**		
1	3.83	**1 (100)**	2 (82.81)	3 (64.99)	84.40	*T. spiralis*
10	4.77	3 (20.85)	**1 (100)**	2 (70.36)	60.0	*T. spiralis*
2	13.86	2 (93.26)	**1 (100)**	3 (74.95)	87.75	*T. spiralis*
15	14.97	3 (70.33)	2 (87.75)	**1 (100)**	72.5	*T. britovi*
11	16.57	**1 (100)**	2 (52.23)	3 (50.92)	88.25	*T. britovi*
3	17.49	3 (75.58)	2 (85.26)	**1 (100)**	74.10	*T. spiralis*
16	21.36	**1 (100)**	2 (92.04)	3 (87.61)	96.55	*T. britovi*
6	23.67	2 (75.28)	**1 (100)**	3 (63.27)	75.65	*T. spiralis*
7	24.68	**1 (100)**	2 (68.02)	3 (20.94)	59.85	*T. spiralis*
4	26.31	**1 (100)**	2 (94.34)	3 (67.07)	67.65	*T. spiralis*
14	28.62	3 (88.28)	2 (94.45)	**1 (100)**	89.40	*T. britovi*
5	29.47	3 (39.89)	**1 (100)**	2 (73.21)	66.85	*T. spiralis*
18	38.81	3 (60.55)	**1 (100)**	2 (93.60)	77.10	*T. britovi*
13	39.78	3 (38.25)	**1 (100)**	2 (78.96)	77.45	*T. britovi*
12	40.16	2 (76.67)	**1 (100)**	3 (66.97)	63.7	*T. britovi*
8	40.34	3 (34.23)	**1 (100)**	2 (74.19)	47.30	*T. spiralis*
17	42.42	3 (27.00)	**1 (100)**	2 (80.11)	70.70	*T. britovi*
9	58.62	2 (77.47)	3 (39.79)	**1 (100)**	53.0	*T. spiralis*

*The ranking data are cross-referenced with the infection level in the entire tongue, tongue mass, and Trichinella species triggering the infection*.

a *Tongue parts are ranked from the highest to the lowest lpg value. The part of the tongue with the highest lpg value is marked in bold*.

**Table 9:** Correlations between the number of larvae per gram (lpg) of the different parts of the tongue in the groups of pigs infected with *T. spiralis* (300 ML/pig) or *T. britovi* (5,000 ML/pig).

**Tongue part**	**Tongue part**						
		**Tip (*****apex lingue*****)**		**Body (*****corpus lingue*****)**		**Root (*****radix linguae*****)**	
		**T1**	**T3**	**T1**	**T3**	**T1**	**T3**
Tip (*apex lingue*)	T1	-	-	0.497	-	0.576	−0.040
	T3	-	-	-	0.080	-	-
Body (*corpus lingue*)	T1	0.497	-	-	-	0.830^*^	-
	T3	-	0.080	-	-	-	0.942^*^
Root (*radix linguae*)	T1	0.576	-	0.830^*^	-	-	-
	T3	-	−0.040	-	0.942^*^	-	-

*Data are R correlation coefficients, calculated using Pearson's or Spearman's correlation as appropriate*.

* *Correlation is significant at the 0.05 level*.

*T1, T. spiralis-infected pigs; T3, T. britovi-infected pigs*.

### Histology of Diaphragm Muscles of Experimentally Infected Pigs

**Figure 2:**
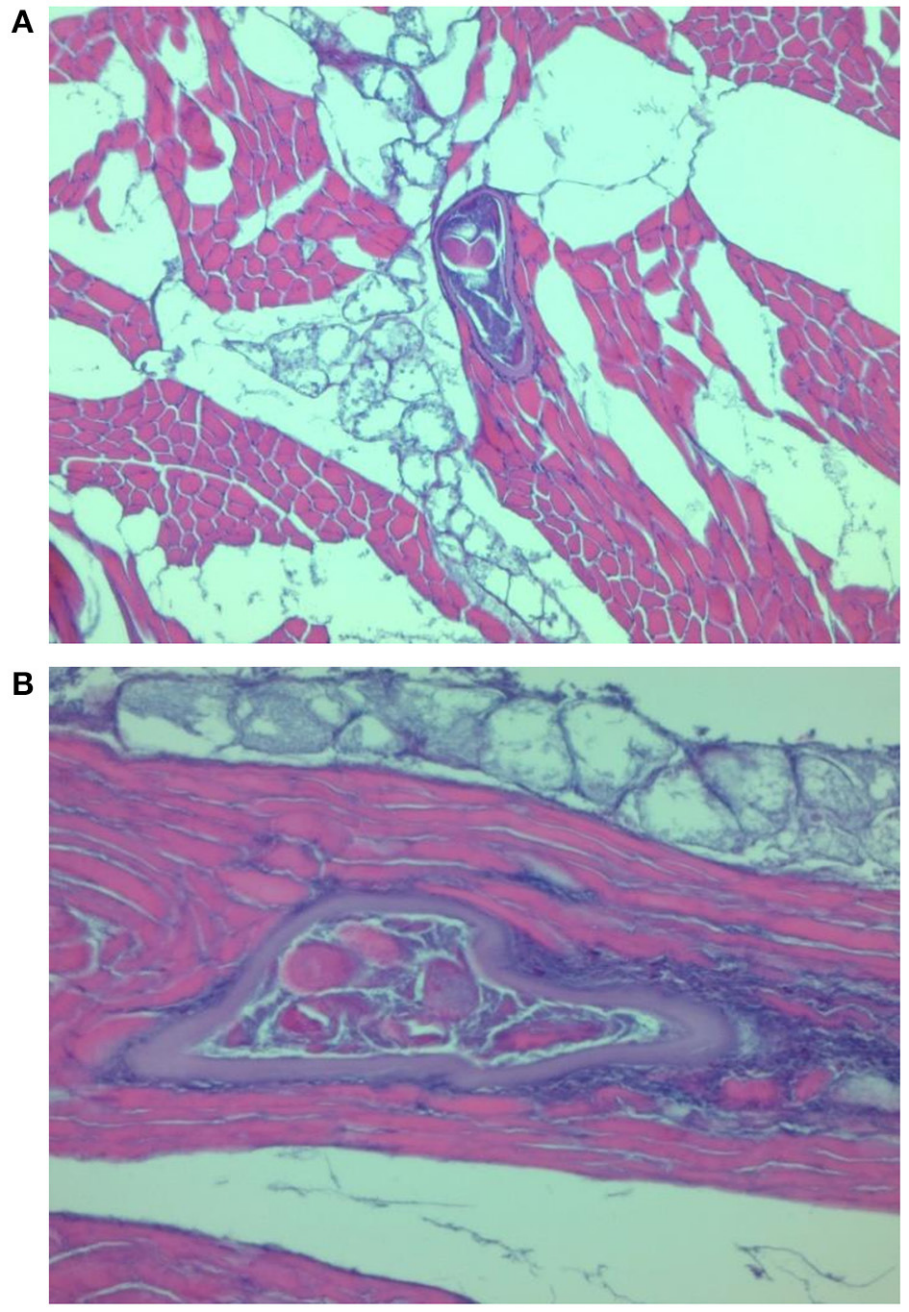
Transformation of muscle cells into a nurse cell. *T. spiralis* larva with a well-formed capsule surrounded by a focal infiltration of inflammatory cells **(A)** and a diffuse inflammatory reaction in the interstitial tissue **(B)**.

**Figure 3:**
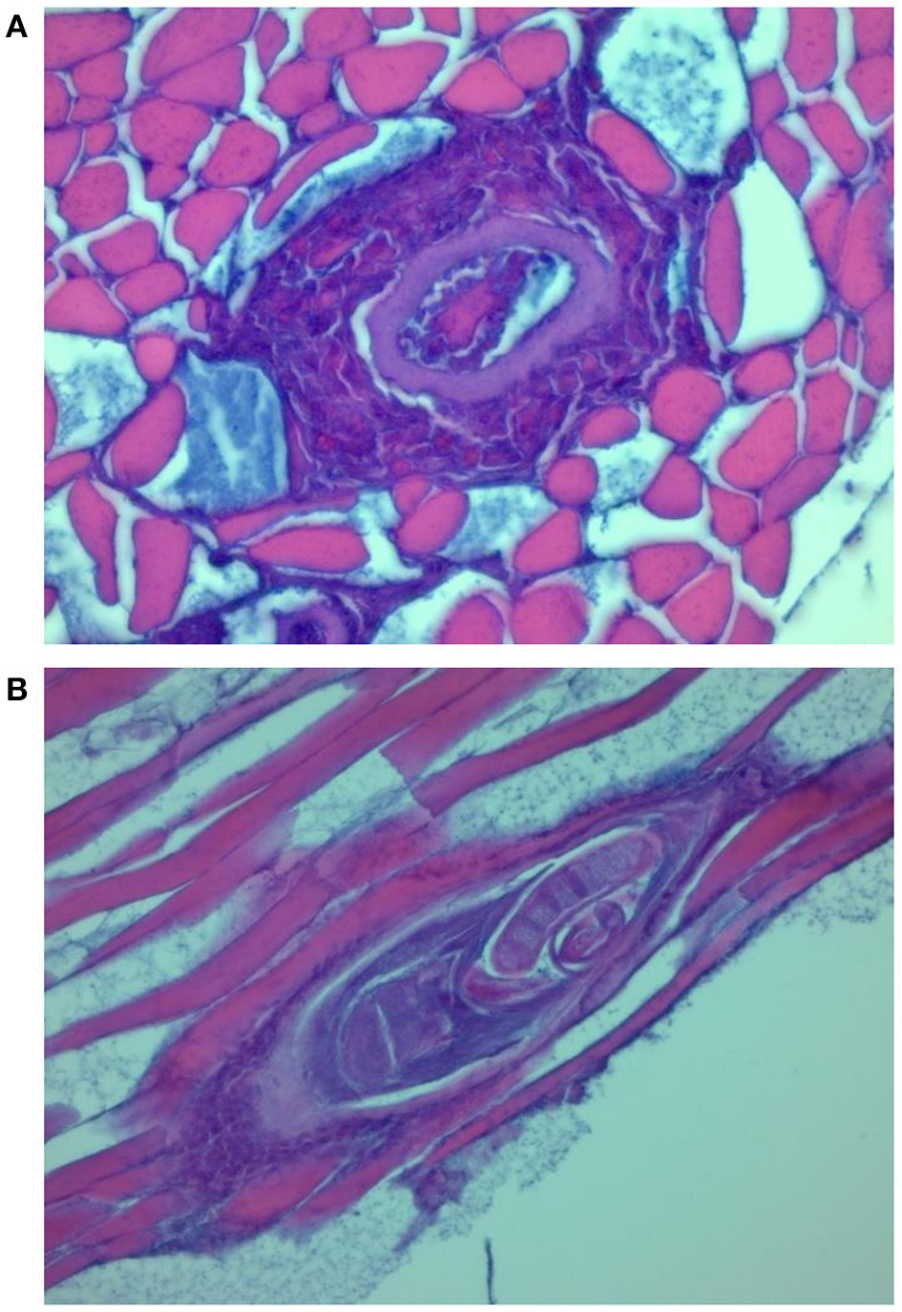
Transverse **(A)** and longitudinal **(B)** section through the nurse cell complex of *T. britovi* larvae (encapsulated). Intense **(A)** or moderate **(B)** cellular infiltrate surrounding the nurse cells.

**Figure 4:**
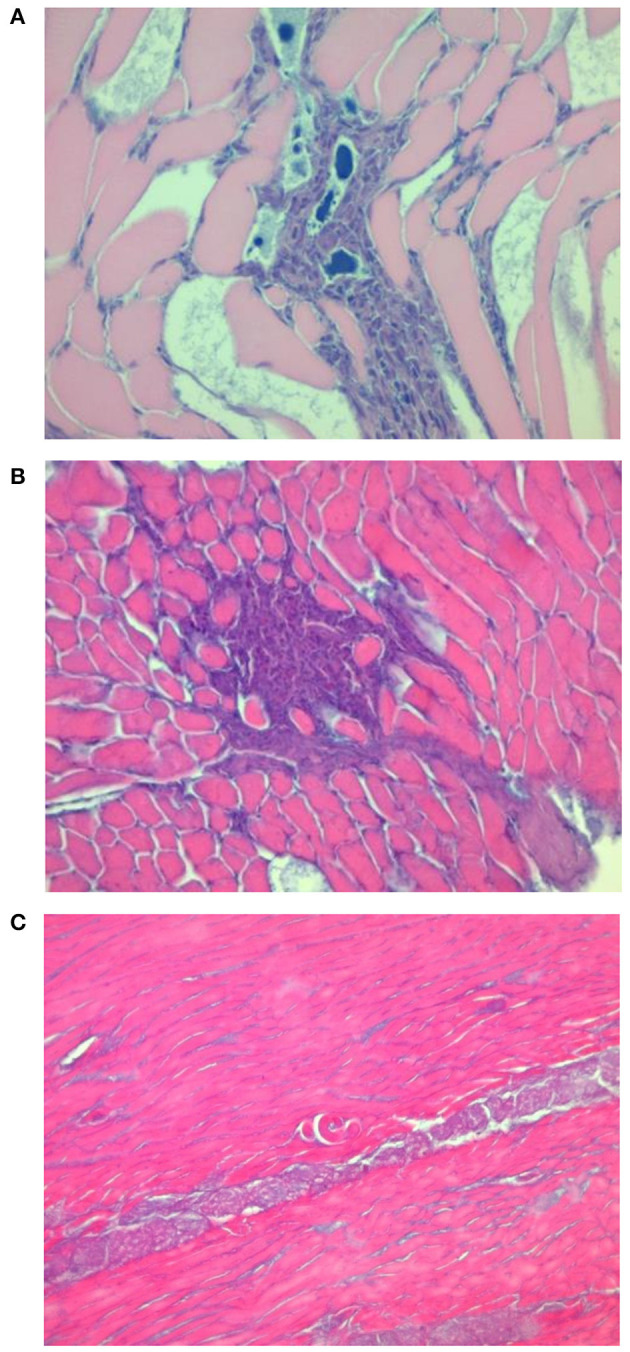
Focal and diffuse mixed inflammatory reaction infiltrates in the muscle tissue of pigs infected with *T. pseudospiralis* **(A,B)**. Single non-encapsulated larva with very mild cell inflammatory reaction in the vicinity of the larvae **(C)**.

Histological examination showed features of a differential inflammatory response around larvae of the different *Trichinella* species. The morphology of samples from pigs infected with *T. spiralis* and *T. britovi* showed a profuse or moderate inflammatory response around the nurse cells (Figures 2A,B, 3A,B) Moreover, in the case of *T. spiralis*, diffuse inflammatory infiltration in the adjacent interstitial tissue was observed more often (Figure 2B). In both cases, the larvae were surrounded by distinct capsules. Muscle tissue of animals infected with *T. pseudospiralis* showed features of focal and diffuse inflammation of varying severity (Figures 4A–C); however, in most samples, the inflammatory reaction was less intense than those caused by *T. spiralis* and *T. britovi* (Figure 4C). The capsule walls were undetectable.

### Confirmation of the Species of *Trichinella* ML Isolated From the Muscles of Infected Pigs

**Figure 5:**
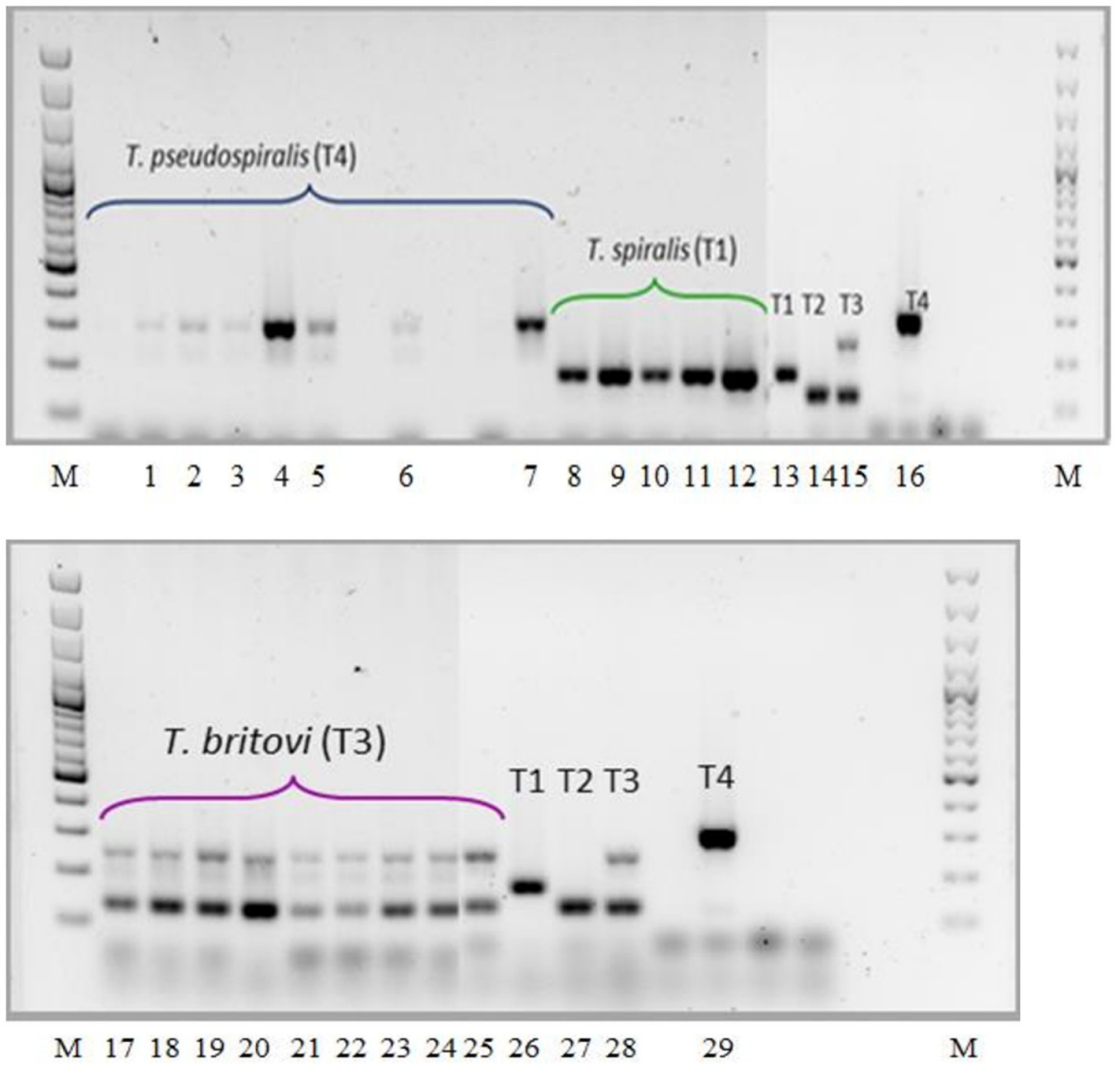
PCR identification of *Trichinella* ML isolated from muscles of experimentally infected pigs. Lane M, molecular marker; lane 1, *T. pseudospiralis* (pig no. 19); lane 2, *T. pseudospiralis* (pig no. 20); lane 3, *T. pseudospiralis* (pig no. 21); lane 4, *T. pseudospiralis* (pig no. 22); lane 5, *T. pseudospiralis* (pig no. 23); lane 6, *T. pseudospiralis* (pigs nos. 24 and 25); lane 7, *T. pseudospiralis* (pigs nos. 26 and 27); lane 8 *T. spiralis* (pigs nos. 1 and 2); lane 9, *T. spiralis* (pigs nos. 3 and 4); lane 10, *T. spiralis* (pigs nos. 5 and 6); lane 11, *T. spiralis* (pigs nos. 7 and 8); lane 12, *T. spiralis* (pigs nos. 9 and 10); lanes 13 and 26, *T. spiralis* ISS003; lanes 14 and 27, *T. nativa* ISS042; lanes 15 and 28, *T. britovi* ISS002; lanes 16 and 29, *T. pseudospiralis* ISS013; lane 17 and 18, *T. britovi* (pig no. 11); lane 19, *T. britovi* (pig no. 12); lane 20, *T. britovi* (pig no. 13); lane 21, *T. britovi* (pig no. 14); lane 22, *T. britovi* (pig no. 15); lane 23, *T. britovi* (pig no. 16); lane 24, *T. britovi* (pig no. 17); lane 25, *T. britovi* (pig no. 18).

As shown in Figure 5, the electrophoretic pattern of the tested *Trichinella* ML isolates obtained from the diaphragms of particular experimental groups of pigs was consistent with that produced by the reference strains, and no contamination (mixed infections) in any of the three groups of swine was observed.

## Discussion

In most European Union countries, slaughtered pigs are officially tested for the presence of *Trichinella* spp. as part of routine post-mortem veterinary examinations of the carcasses. According to Commission Regulation (EU) 2015/1375, several methods based on the digestion of muscle tissue are allowed for this purpose; however, the magnetic stirrer method for pooled-sample digestion is indicated as the reference one (22). In addition to the description of the procedures themselves, the regulation also specifies the pig muscles from which the test sample should be taken as well as the sample mass (22). In the case of fattening pigs and breeding sows or boars, a sample of a minimum weight of 1 or 2 grams, respectively, should be taken from the diaphragm pillars for a pooled-sample digestion assay. When diaphragm pillars are not available, samples of twice the weight should be taken from the costal or sternal part of the diaphragm, or from the tongue, jaw muscles, or abdominal muscles. However, for certain substitute muscles listed in Annex I of Commission Regulation 2015/1375, such as tongue or abdominal muscles, the exact part of the muscle anatomy to be sampled is not specified (22). Furthermore, although the regulation covers the detection of all genotypes of *Trichinella*, there are no systematic data on the distribution of different *Trichinella* species in various parts of the pig diaphragm. In this regard, our studies on the groups of pigs infected with *T. spiralis, T. britovi*, or *T. pseudospiralis* found no significant differences in terms of intensity of *Trichinella* ML infection between the three different parts of the diaphragm, i.e., the pillars, costal, and sternal part. However, in all the experimental groups of pigs, the diaphragm pillars were the most heavily parasitized part. Individual pig results also showed that among these three anatomical regions, the diaphragm pillars exhibited the highest larval burden in 90% of pigs infected with *T. spiralis*, 62.5% of pigs infected with *T. britovi* and 100% of pigs infected with *T. pseudospiralis*. Moreover, in the remaining four pigs in which the highest intensities of *Trichinella* ML infection were observed in parts of the diaphragm other than the pillars, the larval load in the pillars was no lower than 70% of that of the part with the highest intensity. It is widely known that as an animal grows, the proportion by weight of one individual muscle to another or of one part of a given muscle to other parts changes. This phenomenon may induce a dilution of the larvae density and affect the proportionality in terms of larval load between different muscles or different parts of the same muscle because their growth kinetics vary. In this regard, our studies did not provide any significant evidence that differences in the diaphragm weight in the range of 55.80–167.25 g affected the distribution of the larvae, because the mean percentage ratio of the weight of the individual diaphragm parts to the total diaphragm weight in all experimentally infected pigs was rather constant and amounted to 41.82% (±4.61%), 45.44% (±4.50%), and 12.74% (±1.05%) for the pillars, costal, and sternal part, respectively. This may indicate that as the pig's weight and consequently the total mass of the diaphragm increase, the individual parts of the diaphragm (the pillars, costal, and sternal part) show similar growth kinetics expressed as percentage weight gain in relation to the total mass of the diaphragm. It can therefore be assumed that an increase of diaphragm mass will cause the same proportional changes in the intensity of infection in all its three parts.

Our results showed that in the case of pigs infected with encapsulated species of *Trichinella* (*T. spiralis* and *T. britovi*), a positive correlation in terms of larval burden was found between all three parts of the diaphragm, while in the case of pigs infected with *T. pseudospiralis*, such statistical dependencies were shown between the pillars and costal part and between the pillars and sternal part of the diaphragm. It may suggest a slightly different mechanism of the deposition of encapsulated and non-encapsulated *Trichinella* species in the individual parts of the swine diaphragm. Unfortunately, as mentioned above, there are little data available for comparative purposes regarding the distribution of various species of *Trichinella* in diaphragms of infected pigs. Studies conducted by Kotula et al. (20) showed that in pigs infected with 300 ML of *T. spiralis*, the intensity of infection in the left diaphragm pillar was significantly higher than in other parts of this muscle, i.e., the left and right lumbocostal arches, dorsal and ventral sections of the costal part and left and right sides of the sternal part. In addition, the authors also observed these statistical differences for both 2-month-old pigs with lower muscle mass in which the mean intensity of *T. spiralis* infection in the diaphragm pillars was 22.9 lpg and 6-month-old individuals with proportionally higher body weight and mean intensity of *T. spiralis* infection in the pillars of 5.8 lpg (20). Several differences can be indicated between this study and our own. First, the previous researchers digested 12-gram samples (pooling two 1 g and two 5 g samples or, if 5-g samples were impossible to obtain, the infection level for the region was based on the 1 g samples), while in our experiment, entire individual parts of the diaphragm were digested. Furthermore, the authors sorted the pigs into two groups by age and consequently by body weight. This approach allowed more homogeneous groups in terms of infection level. Although the weights of the pigs during both infection and slaughter were aligned in our experiment, we observed a wide variation in the level of infection between individuals, particularly in the group of pigs infected with *T. pseudospiralis* (range: 0.01–65.61 lpg in the diaphragm). As a consequence, this significantly affected the results of both parametric and non-parametric statistics. Our studies also showed that in the group of pigs in which the diaphragm pillars presented the highest intensity of *Trichinella* ML infection, the mean larval density in the costal part amounted to 76.31% and in the sternal part to 75.10% of that observed in the pillars. However, taking into account that the minimum values were 44.45 and 40.68% for the costal and sternal parts, respectively, it is entirely reasonable that for a pooled-sample digestion assay during pig carcass examination, the weight of the specimens from these parts of the diaphragm should be at least double that of specimens from the pillars. This is particularly appropriate when pigs are tested together in the maximum number (25, 50, or 100) specified in Commission Implementing Regulation (EU) 2015/1375 and each pig is represented by a sample mass of 1 (diaphragm pillars from fattening pigs), 2 (the costal or sternal part from fattening pigs or diaphragm pillars from sows or boars), or 4 (the costal or sternal part from sows or boars) grams (22). The theoretical sensitivity of the digestion method is 1 larva per gram; however, as was shown by Prost and Nowakowski (2), at this level of infection in the diaphragm of infected pigs and using 1 gram samples, the actual sensitivity of the method is 73%. This rises to 90 or 100% if, respectively, a minimum of 2 and 3 grams of muscle tissue are tested (2). Similarly, Forbes and Gajadhar (8) showed that the larval loads of 1.0–1.9 lpg require 3–5 g samples of muscle tissue for reliable detection. Therefore, in order to fully understand the interdependence in the density of the larvae of particular *Trichinella* species between the pillars and the remaining two parts of the diaphragm, further studies are needed, and to best serve food safety and adhere to the principles of the pooled-sample digestion method described in Regulation 1375/2015, the level of infection in the diaphragm pillars of pigs experimentally infected with various species of *Trichinella* would be 1 larva per gram of tissue (the detection limit). In such a scenario, the parts of the diaphragm should be divided into individual 1 g (diaphragm pillars) and 2 g (costal and sternal parts) tissue specimens and then digested. However, generating an infection of as low density as 1 larva per gram of diaphragm pillar tissue in pigs is difficult. It would require a large number of experimental animals, many combinations of low doses for each species of *Trichinella* or, possibly, skillful control of the phases of infection (time from infection to slaughter of the pigs). This control might be shortening the duration of the intestinal phase (slaughtering the animals and thereby reducing the number of newborn larvae migrating to the muscles) or extending the muscle phase (prolonging the duration of the infection and thereby reducing the number of larvae in the muscles). In this context, some important data were provided by Kotula et al. (20) who showed that in the case of *T. spiralis* infection in pigs, 1 gram samples taken from the diaphragm pillars generated a lower percentage of false negative results (8.5%) than samples obtained from other diaphragm parts (13.3%).

Our studies also showed that the distribution of *Trichinella* ML in pig diaphragms may be partially dependent on their level in the entire diaphragm. In the group of pigs with moderate (21–35 lpg) or moderately high (35–55 lpg) whole-diaphragm intensity of *Trichinella* spp. ML infection, the larval density in the pillars was significantly higher than that in the costal or sternal part. On the other hand, no such differences were observed within the groups of pigs presenting a low (4–12 lpg), moderately low (12–21 lpg) or high (>60 lpg) level of *Trichinella* spp. infection. This phenomenon is difficult to explain; it could have been influenced by hemodynamic differences in the diaphragm muscle as responses to different numbers of penetrating larvae. The influence of the intensity of *Trichinella* larvae infection on predilection sites in *Trichinella*-infected swine was also demonstrated by Serrano et al. (21). The authors infected pigs with different doses (25-36000) of *T. spiralis* or *T. britovi* ML and found that in heavily infected swine (146–3,634 lpg) the highest intensity of infection was observed in the diaphragm pillars, while in light or moderate infections the base of the tongue showed higher larval densities. Unfortunately, the authors did not provide data to indicate whether the infection level had any impact on *Trichinella* ML distribution in particular parts of the diaphragm; however, they did find that in lightly infected pigs, the diaphragm pillars presented higher larval density than the lumbocostal, costal, and sternal parts.

Our research showed that both in groups of pigs infected with particular species of *Trichinella* as well as groups with different levels of infection, the sampling site had no significant effect on the observed intensity of infection. This may be of particular importance in epidemiological and experimental studies. In other words, in experiments in which the determination of the larval burden in infected swine is an additional procedure and not the main goal of the study, it is sufficient to take a sample from one side of an individual part of the diaphragm muscle to obtain viable results in reasonable examination time and when sample availability is possibly limited.

Another aim of this study was to assess the distribution of *Trichinella* ML in different sections of the tongue. Unfortunately, tongues from pigs infected with *T. pseudospiralis* were intended for other research, hence results were obtained only for pigs infected with *T. spiralis* and *T. britovi*. In this study no statistically significant differences were found between the tip, body and root of the tongue in pigs infected with *T. spiralis* and those infected with *T. britovi*. Individual pig results showed that regardless of the species of *Trichinella* used for experimental infection, the body of the tongue most often showed the highest intensity of infection (in 50% of all infected animals). In the remaining 50% of infected swine, the tip or tongue root was the most intensely infected. It should be concluded, therefore, that based on these studies, it is difficult to indicate a distinct distribution pattern of encapsulated *Trichinella* genotypes in the tongues of infected pigs. However, in both *T. spiralis*- and *T. britovi*-infected pigs, a positive correlation in larval burden was found between the body and the root of the tongue. Kapel et al. (7) demonstrated that in pigs infected with 10,000 ML of *T. spiralis* or *T. britovi*, the tongue tip showed higher intensity of infection than the tongue base in all eight infected individuals. Other comprehensive studies conducted by Kapel et al. (19) showed that in pigs and wild boars infected with encapsulated (i.e., *T. spiralis, T. nativa, T. britovi*, and *T. nelsoni*) and non-encapsulated (*T. pseudospiralis*) *Trichinella* genotypes, the tongue base presented a higher larval burden than the tip. Our study also facilitates comparison of infection levels in parts of the tongue with those in parts of other muscles, and it should also be highlighted that the mean larval density of the tongue part by part by part was 51.64% for the tip (range: 8.99–116.13%), 63.97% for the base (range: 28.64–106.89%), and 56.18% for the root (range: 11.53–101.75%) of the tongue of that observed in the diaphragm pillars (data not shown). In this context, special attention should be paid to the minimum values, which indicated that in some individuals the intensity of *Trichinella* ML infection in the tongue was even ten times lower than that in the diaphragm pillars. This may suggest that in pig carcass examination, the 2 gram sample taken from the tongue tip or root should not always be considered equivalent to the 1 gram sample taken from the diaphragm pillars.

Histological changes observed in the diaphragm muscle tissue of pigs infected with *T. spiralis* and *T. britovi* were typical and comparable to those observed by Gamito-Santos et al. (24) in skeletal muscles of experimentally infected wild boars; however, those authors showed much stronger inflammatory reactions in samples from wild boars infected with *T. spiralis* than in those inoculated with *T. britovi*. Our study also confirmed that diffuse inflammatory infiltration in the adjacent interstitial tissue was observed more often in muscles of pigs infected with *T. spiralis* than with *T. britovi*. The inflammatory reaction was significantly less intense in the diaphragm muscles of pigs infected with *T. pseudospiralis*. These observations may partially indicate that *T. pseudospiralis* shows a lower pathogenicity for pigs than the encapsulated species of *Trichinella*, i.e., *T. spiralis* and *T. britovi*. A weaker inflammatory response was also shown in the skeletal muscles of mice infected with *T. pseudospiralis* as compared to the response in encapsulated species–infected mice skeletal muscle in several other experiments which introduced classical histological techniques, immunohistochemistry or immunoblotting (25–31).

In conclusion, this is the first attempt to assess the distribution of different species of *Trichinella* in various parts of the diaphragm of experimentally infected pigs. The diaphragm pillars were the most heavily parasitized part of the diaphragm both in groups of pigs infected with particular species of *Trichinella* (i.e., *T. spiralis, T. britovi*, and *T. pseudospiralis*) and in groups of pigs presenting different levels of infection; however, statistical differences were observed only in the group of pigs with moderate or moderately high whole-diaphragm intensity of *Trichinella* spp. infection. In all groups of pigs, regardless of the infecting *Trichinella* species or infection level, larvae showed a homogeneous distribution on both sides of the diaphragm. When larval density determination is performed as a complementary procedure and is not the main goal of an experiment, and in epidemiological studies, it is sufficient to take a sample from only one part and either side of the diaphragm without distinction. This study also confirmed that official fattening pig carcass examinations using a pooled-sample digestion assay on 1-gram samples taken from diaphragm pillars, should, if the diaphragms pillars are not available, use samples taken from the remaining diaphragm parts (costal or sternal) of at least double the mass of samples from the pillars. Further studies using pigs in which the larvae density of various *Trichinella* species would be at the theoretical detection limit of the pooled-sample digestion method (i.e., 1 larva/gram of tissue) are necessary to fully appraise the relationship between the pillars and the other parts of the diaphragm in terms of intensity of *Trichinella* ML infection. Muscle larvae of both *T. spiralis* and *T. britovi* showed a homogeneous distribution in all three parts of the tongue; however, individual pig results may suggest that a sample taken from the root or tip of the tongue with twice the weight may not be equivalent to a sample taken from the diaphragm pillars in terms of detection of trichinellosis induced by encapsulated species of *Trichinella* in pigs. Histological findings also confirmed that the inflammatory pattern of pig muscles varies depending on the *Trichinella* species triggering the infection and is less intense in infections induced by *T. pseudospiralis* than in those caused by encapsulated species of *Trichinella* (*T. spiralis* and *T. britovi*).

## Data Availability Statement

The raw data supporting the conclusions of this article will be made available by the authors, without undue reservation.

## Ethics Statement

The study was approved by the Second Local Ethics Committee for Animal Experimentation at the University of Life Sciences in Lublin (resolutions nos. 34/2013, 4/2014, and 77/2015).

## Author Contributions

MG: conceptualization, methodology, funding acquisition, writing—original draft, project administration, data curation, and resources. PK: methodology. RP-Ł: writing—review editing. AŁ and AK: methodology. MP-M: methodology and writing—review editing. All authors read and approved the manuscript.

## Conflict of Interest

The authors declare that the research was conducted in the absence of any commercial or financial relationships that could be construed as a potential conflict of interest.
