# Supplementary material for: Distribution of Trichinella spiralis, Trichinella britovi, and Trichinella pseudospiralis in the Diaphragms and T. spiralis and T. britovi in the Tongues of Experimentally Infected Pigs
Source: Front Vet Sci. 2021 Jun 22;8:696284. doi: 10.3389/fvets.2021.696284 (PMC8258146; doi:10.3389/fvets.2021.696284)
Supplement: Supplementary file 1 [file Data_Sheet_1.pdf]

Supplementary Material

1.1 Supplementary Figures

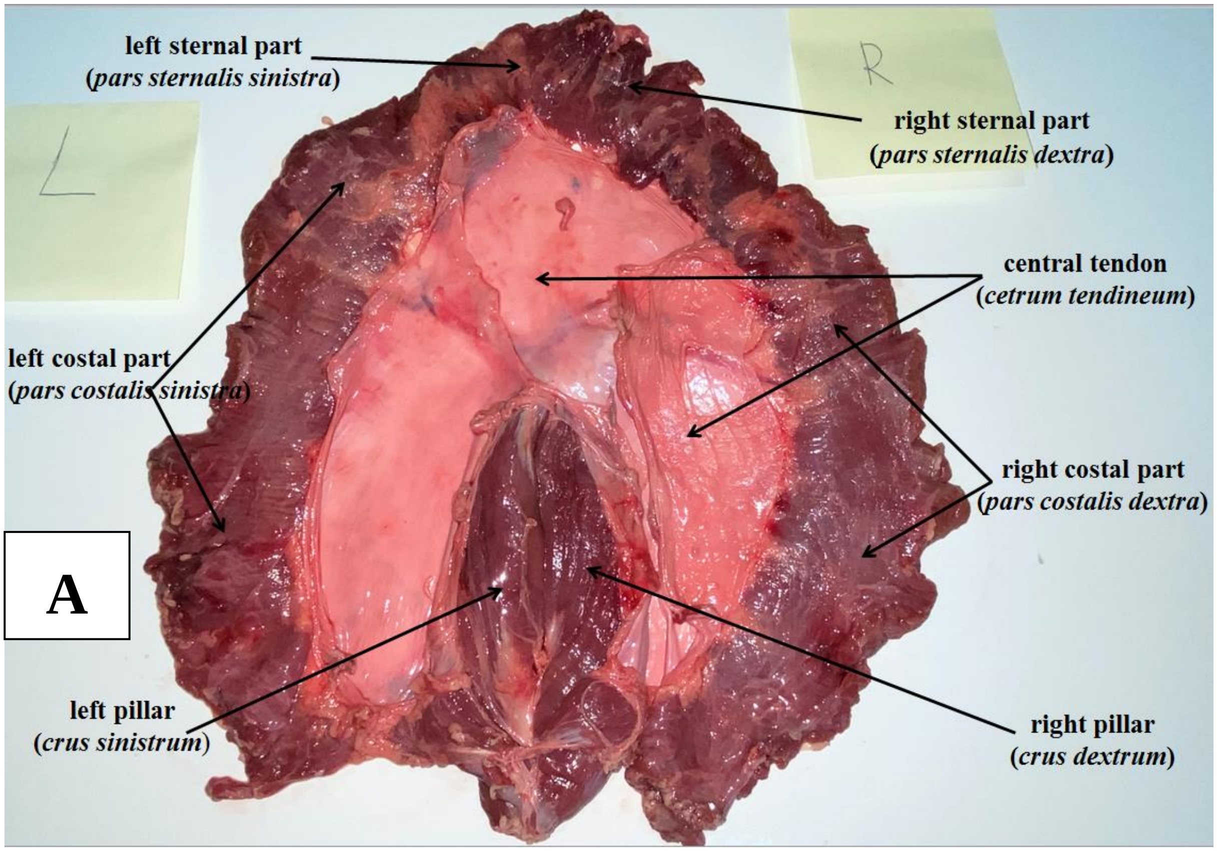

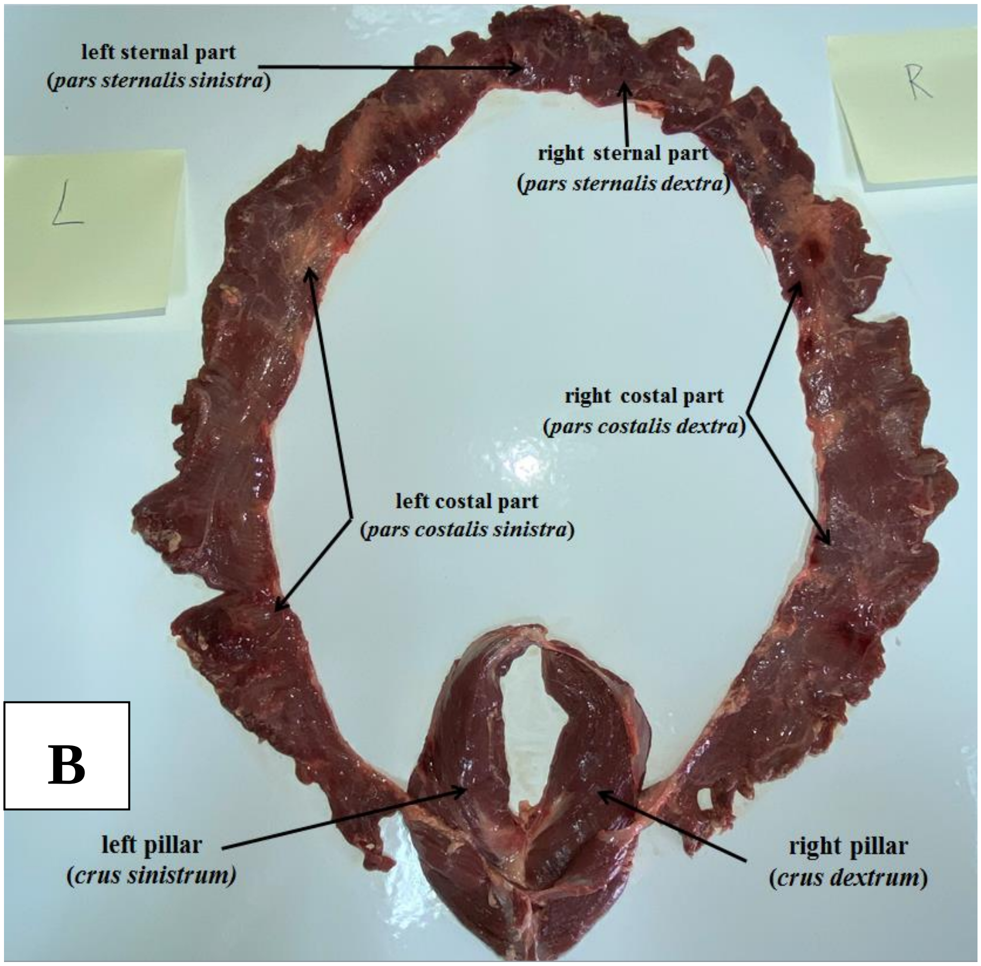

Supplementary Figure 1. The entire diaphragm with central tendon immediately after its removal form the infected pigs **(A)** and the entire diaphragm without central tendon dissected and prepared for examination **(B)**. Each part (the pillars, costal part and sternal part) of each side is marked with arrows.

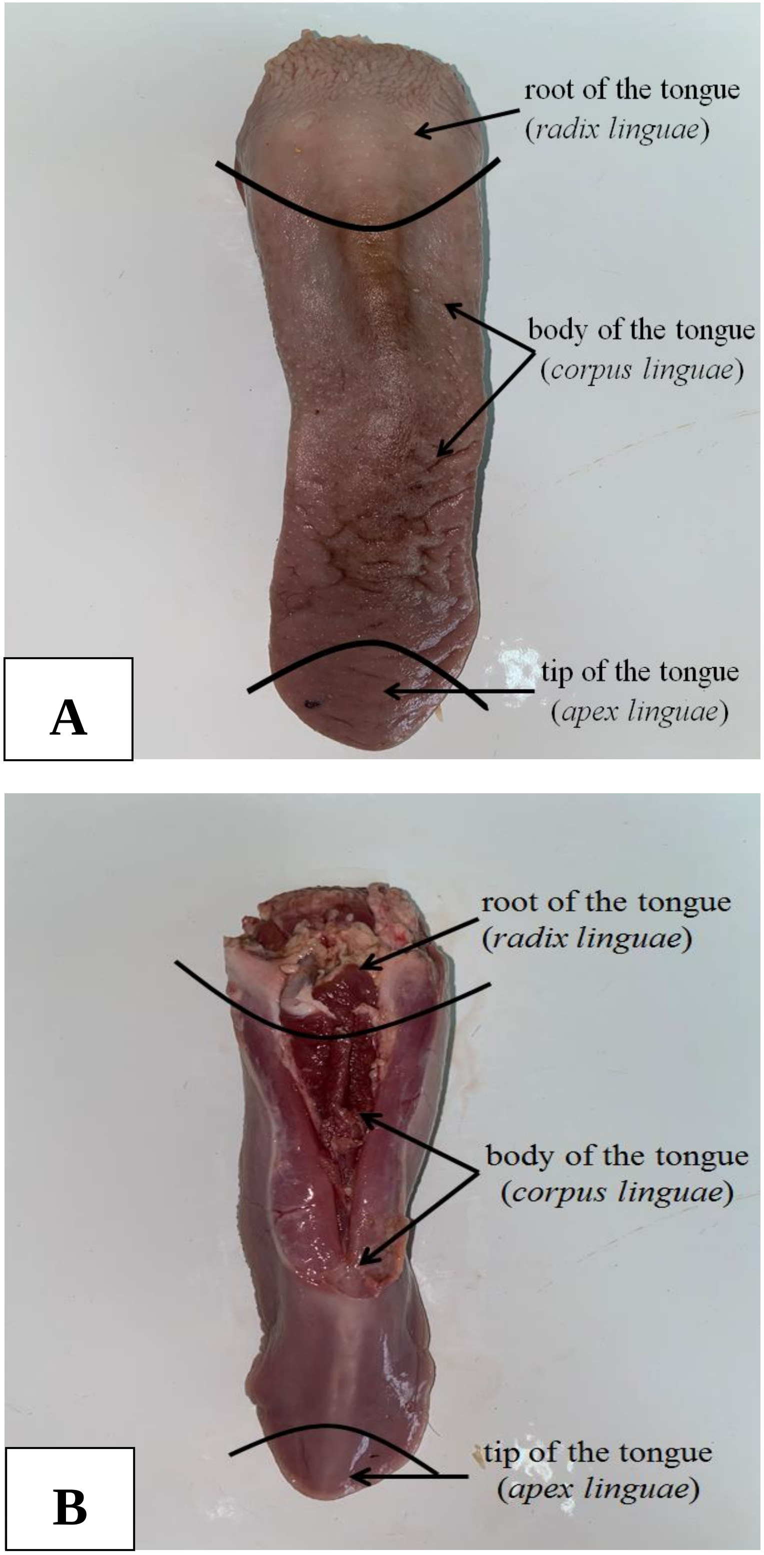

Supplementary Figure 2. The entire tongue immediately after its removal from the infected pigs: dorsal **(A)** and ventral **(B)** surface. Each part (the tip, body and root) is marked with arrows.
